# Association of frailty with health service use among older Chinese adults: analysis of population-based panel data

**DOI:** 10.3389/fpubh.2023.1011588

**Published:** 2023-07-28

**Authors:** Rui Yan, Lifeng Li, Xiaoran Duan, Jie Zhao

**Affiliations:** ^1^Internet Medical and System Applications of National Engineering Laboratory, The First Affiliated Hospital of Zhengzhou University, Zhengzhou, Henan, China; ^2^Department of Oncology, The First Affiliated Hospital of Zhengzhou University, Zhengzhou, Henan, China

**Keywords:** frailty, health service use, older adults, panel data, CHARLS

## Abstract

**Background:**

Frailty is a common syndrome characterized by rapid growth in the aging population that has an impact on healthcare systems. This study aimed to investigate the impact of frailty on health service use and whether this effect varies with chronic diseases and socioeconomic status among older individuals in China.

**Methods:**

A balanced panel data analysis was conducted on 3,306 older individuals who completed follow-ups for the three waves of the China Health and Retirement Longitudinal Study (CHARLS) in 2011, 2013, and 2015. The Physical Frailty Phenotype (PFP) Scale was used to assess frailty status. Negative binomial regression was used to test the associations between frailty status, outpatient visits in the past 4 weeks, and annual inpatient hospital days.

**Results:**

Compared with robust individuals, individuals with pre-frail or frail status were likely to report a higher number of outpatient visits [pre-frail: incidence rate ratio (IRR) = 1.28, 95% CI = 1.16–1.41; frail: IRR = 1.45, 95% CI = 1.23–1.71], and inpatient hospital days (pre-frail: IRR = 1.40, 95% CI = 1.24–1.58; frail: IRR = 2.17, 95% CI = 1.81–2.60) after controlling for all covariates. All five frailty components (weight loss, exhaustion, low physical activity, slowness, and weakness) were associated with a higher number of inpatient hospital days, and two components (weight loss and exhaustion) were associated with a higher number of outpatient visits. The effect of frailty on inpatient hospital stays persisted in different socioeconomic groups, across all health insurance programmes and physical comorbidities.

**Conclusion:**

Frailty is associated with greater health service use among older individuals. Effective screening, prevention, intervention, and management of frailty may be important to reduce health service use.

## 1. Introduction

The World Population Prospects 2019 highlighted that the world's population is growing older (1). It is estimated that one in six people will be aged 60 years or older by 2030 (2). Population aging worldwide has major implications for healthcare systems. Aging is associated with an increase in the frequency and severity of several chronic diseases, such as cardiovascular diseases, diabetes, chronic respiratory diseases, and cancer. Two in five middle-aged and older Chinese individuals live with more than one or multiple chronic conditions (3), resulting in a great burden on health service use. Given the rapidly aging population in China, there is a great need to understand the factors that influence the utilization of health services by older individuals to reduce related costs for individuals and society.

Frailty has become a challenge and has drawn increasing attention with the rapid growth of the aging population. Frailty is a common syndrome characterized by age-related physiological decline and vulnerability to poor homeostasis following stress (4, 5). A recently published systematic review including 1,755,497 participants from 62 countries and territories indicated a global pooled physical frailty prevalence of 12% and a pooled pre-frailty prevalence of 46% (6). Physical, psychological, cognitive, and social factors contribute to frailty (7). Previous studies have indicated that poor physical health, cognitive dysfunction, depressive symptoms, and reduced health perception are associated with a high risk of frailty (8–10). Frailty reflects multisystem physiological changes (7). It has become an important and valuable measure for evaluating the health status of older adults and is an emerging public health priority (11).

Previous studies have found that frailty is associated with increased healthcare utilization in China (12, 13). Tian et al. (12) conducted a cross-sectional questionnaire survey among 915 older adults and found that frail older adults had higher odds of hospitalization and emergency use. Gao et al. (13) found that participants who were frail were more likely to report higher odds of outpatient visits and inpatient visits by using baseline data (2011–2012) of older adults from the China Health and Retirement Longitudinal Study (CHARLS). These published studies investigating the implications of frailty on health service use in China evaluated small sample sizes (12) and used cross-sectional designs (13). Therefore, it is necessary to determine the effect of frailty status on health-service use using Chinese data from a national longitudinal analysis. Additionally, most previous studies on the association between frailty status and health-service use only analyzed the presence of frailty as a tripartite variable (robust, pre-frail, and frail) without considering the contribution of each component of frailty to health-service use (12, 13), which gives us the impetus to examine the association between specific components of frailty and health-service use. Finally, given that chronic diseases and socioeconomic status are both associated with frailty (14–16) and health service use (17–20), chronic diseases and socioeconomic status may be important confounders between frailty and health service use. Whether the impact of frailty on health service use varies according to chronic disease and socioeconomic status remains unclear. It is important to identify a separate association between frailty and health service use by stratifying older adults into different groups according to their comorbid chronic diseases and socioeconomic status. Insights into these associations will provide targeted information for health policymaking and improve the promotion of health among older people.

The current study aimed to determine the effect of frailty on health service use by examining unique elements of healthcare use, such as the number of outpatient visits in the past 4 weeks and inpatient hospital days during the past year, among older individuals (aged ≥ 60 years) from 2011 to 2015 in China. National representative data were obtained from the China Health and Retirement Longitudinal Study (CHARLS) (21). We hypothesized that frailty status and specific frailty components are associated with increased health service use in older Chinese individuals.

## 2. Materials and methods

### 2.1. Study population

This current population-based cohort study was nested within the China Health and Retirement Longitudinal Study (CHARLS) (21). The CHARLS enrolled participants from 450 urban communities and rural areas in 28 provinces in China using multistage stratified probability-proportional-to-size sampling (21). The follow-up survey was carried out every 2 years after the baseline survey in 2011. The second- and third-wave surveys were conducted in 2013 and 2015, respectively. Self-reported data on sociodemographic factors, lifestyle behaviors, and chronic disease status were collected from all the participants using a standardized questionnaire.

A total of 17,708 participants were enrolled in the baseline survey conducted in 2011. We then excluded participants younger than 60 years of age, those lost to follow-up in any of the following two waves, or those with missing data on frailty, health service use, or confounding factors. As a result, 3,306 participants were included in the balanced panel data analysis. The CHARLS study was approved by the Biomedical Ethics Review Committee of Peking University (No. IRB00001052-11015), and informed consent was obtained from all participants in the CHARLS study.

### 2.2. Assessment of frailty and pre-frailty

The Physical Frailty Phenotype (PFP) (22) scale was used to assess frailty and pre-frailty. The PFP consists of five elements: weight loss, exhaustion, low physical activity, slowness, and weakness. Weight loss refers to more than 5 kg or at least 5% unintentional weight loss compared with the previous year's body weight. Exhaustion was identified using two statements from the Centre for Epidemiologic Studies Depression Scale (CES-D) (23): (a) I felt that everything I did was an effort, and (b) I could not get going. These two questions were asked in the form of “How often in the last week did you feel this way: rarely or none of the time (<1 day), some or a little of the time (1–2 days), occasionally or a moderate amount of the time (3–4 days), and most or all of the time (5–7 days).” Subjects who answered at least 3 days to either of the two questions regarding exhaustion were classified as “frail.” “Low physical activity” was defined as the respondent being unable to walk for at least 10 min in an ordinary week. “Slowness” was based on the time needed to finish a 2.5-m distance walk, adjusted for gender and height; the slowest 20% of the respondents were defined as “slow.” “Weakness” was based on the respondents' maximum hand grip strength, adjusting for gender and body mass index (BMI); the lowest 20% of the respondents were defined as “weak.” Individuals who met none of the five criteria were classified as “robust,” those who met one or two criteria were “pre-frail,” and those who met at least three criteria were “frail.”

### 2.3. Health service use

Health service use included the number of outpatient visits in the last 4 weeks and inpatient hospital days during the past year. The number of outpatient visits refers to the visits to medical facilities for outpatient treatment in the past month (excluding hospitalisations). The types of medical facilities included general, specialized, Chinese medicine, township hospitals, community healthcare centers, healthcare posts, and village or private clinics. Inpatient hospital days refer to the number of days of hospitalization (inpatient care) in the past year.

### 2.4. Covariates

We included the following variables as covariates: age, gender, marital status (married/partnered, separated/divorced/widowed/never married), education (no formal education/illiterate, primary school, middle school, and above), residence (rural or urban), smoking status (never smoked, former smoker, or current smoker), and drinking status (never, <once/month, or ≥once/month). Participants were asked to indicate either “yes” or “no” on a list of physician-diagnosed chronic diseases, including hypertension, dyslipidaemia, diabetes or high blood sugar, cancer or malignant tumor, chronic lung diseases, liver disease (except fatty liver, tumors, and cancer), heart disease, stroke, kidney disease (except for tumor or cancer), stomach or other digestive diseases (except for tumor or cancer), and arthritis or rheumatism. The total number of chronic diseases was classified as “0,” “1,” “2,” “3,” or “≥4.”

Health insurance types were classified into four groups: no insurance, urban employee basic medical insurance (UEBMI), urban resident basic medical insurance (URBMI), and a new rural cooperative medical scheme (NRCMS). In each survey wave, annual per-capita household consumption expenditure was split into quartiles to indicate participants' socioeconomic status [quartile 1 (lowest), quartile 2, quartile 3, and quartile 4 (highest)]. According to a previous study, the economic development regions were classified into five groups according to the gross domestic product per-capita income at the province level in China {group 1 [(most affluent)], >$12,000; group 2, $12,000 to >10,000; group 3, $10,000 to >7,000; group 4, $7,000 to >6,000; and group 5 [most deprived], ≤ $6,000} (24).

### 2.5. Statistical analysis

Means and standard deviations were calculated for continuous variables, and numbers and percentages were calculated for categorical variables. Baseline characteristics were summarized according to frailty status and compared between participants grouped as robust, pre-frail, and frail using the one-way ANOVA or the chi-square test, as appropriate. A one-way ANOVA was also conducted to compare the number of outpatient visits and inpatient hospital days between the different sociodemographic groups and frailty statuses.

Using a panel data approach, random-effects negative binomial regression models were used to evaluate the incidence rate ratio (IRR) and 95% confidence intervals (95% CIs) of the impact of frailty status on the number of outpatient visits and inpatient hospital days. Crude IRRs (95% CI) were calculated using the crude model without any adjustments to Model 1. Four further models were fitted: in Model 2, age and sex were adjusted; in Model 3, age, sex, marital status, educational level, residence status, socioeconomic status quartiles, health insurance type, and economic development regions were adjusted; in Model 4, smoking status and drinking frequency were entered while retaining all the covariates of Model 3; and in Model 5, the number of chronic non-communicable diseases was entered while retaining all the covariates of Model 4. Subgroup analyses were performed to examine whether the effect of frailty status on health service use was moderated by socioeconomic status, health insurance type, number of comorbidities, and history of each chronic non-communicable disease. Restricted cubic spline regression models fitted for random effects and negative binomial regression with four knots at continuous PFP scores were further used to explore potential non-linear associations. Sensitivity analyses were conducted using the Poisson regression model with a robust standard error (25) instead of the negative binomial regression model to evaluate the association between frailty status, outpatient visits, and inpatient hospital days.

All statistical analyses in our study were performed using the Stata statistical software version 15.0 and the R statistical software version 4.0.2. Statistical significance was set at a two-sided *P*-value of < 0.05.

## 3. Results

### 3.1. Baseline characteristics of participants

The baseline characteristics of the eligible study participants in 2011 (*N* = 3,306) are presented in Table 1. The mean age was 66.88 years (standard deviation, 5.44) in 2011. Among the 3,306 participants (1,527 men and 1,779 women), most were married/partnered (83.09%), 65.82% lived in rural areas, and 29.82% had no formal education or were illiterate. Most participants (94.79%) had health insurance, and 2,968 (89.77%) were enrolled in the UEBMI, URBMI, or NRCMS. Furthermore, 33.48% were current smokers, 26.65% consumed alcohol at least once per month, and 82.99% had at least one physical comorbidity (Table 1).

**Table 1:** Baseline characteristics of 3,306 participants according to the frailty status.

**Characteristics**	**Total**	**Robust**	**Pre-frail**	**Frail**	***P***
	***N*** **(%)**	***n*** **(%)**	***n*** **(%)**	***n*** **(%)**	
All participants	3,306	1,147 (34.69%)	1,939 (58.66%)	220 (6.65%)	
Age, mean ± SD, years	66.88 ± 5.44	66.12 ± 4.95	67.09 ± 5.53	69.02 ± 6.42	< 0.001
**Age (years)**					
60–70	2,459 (74.38%)	919 (80.12%)	1,411 (72.77%)	129 (58.64%)	
70–80	762 (23.05%)	211 (18.40%)	474 (24.45%)	77 (35.00%)	
80 and above	85 (2.57%)	17 (1.48%)	54 (2.78%)	14 (6.36%)	< 0.001
**Gender**					
Male	1,527 (46.19%)	514 (44.81%)	912 (47.03%)	101 (45.91%)	0.487
Female	1,779 (53.81%)	633 (55.19%)	1,027 (52.97%)	119 (54.09%)	
**Education**					
No formal education/illiterate	986 (29.82%)	270 (23.54%)	623 (32.13%)	93 (42.27%)	< 0.001
Primary school	1,673 (50.6%)	579 (50.48%)	987 (50.90%)	107 (48.64%)	
Middle school and above	647 (19.57%)	298 (25.98%)	329 (16.97%)	20 (9.09%)	
**Marital status**					
Married and partnered	2,747 (83.09%)	979 (85.35%)	1,598 (82.41%)	170 (77.27%)	0.006
Unmarried and others	559 (16.91%)	168 (14.65%)	341 (17.59%)	50 (22.73%)	
**Socioeconomic group**					
Quartile 1 (lowest)	827 (25.02%)	240 (20.92%)	521 (26.87%)	66 (30.00%)	< 0.001
Quartile 2	828 (25.05%)	271 (23.63%)	493 (25.43%)	64 (29.09%)	
Quartile 3	825 (24.95%)	288 (25.11%)	487 (25.12%)	50 (22.73%)	
Quartile 4 (highest)	826 (24.98%)	348 (30.34%)	438 (22.59%)	40 (18.18%)	
**Residence status**					
Urban	1,130 (34.18%)	482 (42.02%)	593 (30.58%)	55 (25.00%)	< 0.001
Rural	2,176 (65.82%)	665 (57.98%)	1,346 (69.42%)	165 (75.00%)	
**Economic development region**					
Group 1 (most affluent)	547 (16.55%)	245 (21.36%)	274 (14.13%)	28 (12.73%)	< 0.001
Group 2	580 (17.54%)	198 (17.26%)	327 (16.86%)	55 (25.00%)	
Group 3	1,548 (46.82%)	515 (44.90%)	941 (48.53%)	92 (41.82%)	
Group 4	376 (11.37%)	127 (11.07%)	218 (11.24%)	31 (14.09%)	
Group 5 (most deprived)	255 (7.71%)	62 (5.41%)	179 (9.23%)	14 (6.36%)	
**Health insurance**					
None	172 (5.21%)	59 (5.14%)	101 (5.21%)	12 (5.45%)	< 0.001
UEBMI	372 (11.25%)	179 (15.61%)	180 (9.28%)	13 (5.91%)	
URBMI and NRCMS	2,596 (78.52%)	829 (72.28%)	1,575 (81.23%)	192 (87.27%)	
Others	166 (5.02%)	80 (6.97%)	83 (4.28%)	3 (1.36%)	
**Smoking status**					
Never	1,840 (55.66%)	650 (56.67%)	1,077 (55.54%)	113 (51.36%)	0.623
Former	359 (10.86%)	126 (10.99%)	209 (10.78%)	24 (10.91%)	
Current	1,107 (33.48%)	371 (32.35%)	653 (33.68%)	83 (37.73%)	
**Drinking frequency**					
Never	2,226 (67.33%)	733 (63.91%)	1,324 (68.28%)	169 (76.82%)	0.001
< 1/month	199 (6.02%)	85 (7.41%)	104 (5.36%)	10 (4.55%)	
≥once/month	881 (26.65%)	329 (28.68%)	511 (26.35%)	41 (18.64%)	
**Number of comorbidities**					
None	893 (27.01%)	360 (31.39%)	482 (24.86%)	51 (23.18%)	< 0.001
One	1,021 (30.88%)	370 (32.26%)	599 (30.89%)	52 (23.64%)	
Two	718 (21.72%)	236 (20.58%)	439 (22.64%)	43 (19.55%)	
Three	384 (11.62%)	113 (9.85%)	229 (11.81%)	42 (19.09%)	
Four and above	290 (8.77%)	68 (5.93%)	190 (9.80%)	32 (14.55%)	

SD, standard deviation; UEBMI, urban employee basic medical insurance; URBMI, urban resident basic medical insurance; NRCMS, new rural cooperative medical scheme. Others include government medical insurance, private medical insurance, and other medical insurances.

In our study, 220 (6.65%) participants were frail, and 1,939 (58.66%) participants were pre-frail. The proportions of older participants with an unmarried status living in rural residences, with lower education levels, from more deprived economic development regions, and lower socioeconomic groups in participants with frailty were higher than those in robust participants (all *p* < 0.05). The proportion of participants with a greater number of comorbidities was also higher among frail participants than among robust participants. For example, in 2011, the proportions of participants with at least four comorbidities were 5.93, 9.80, and 14.55% among the robust, pre-frail, and frail participants, respectively (Table 1).

### 3.2. Association between frailty and health service use

**Figure 1:**
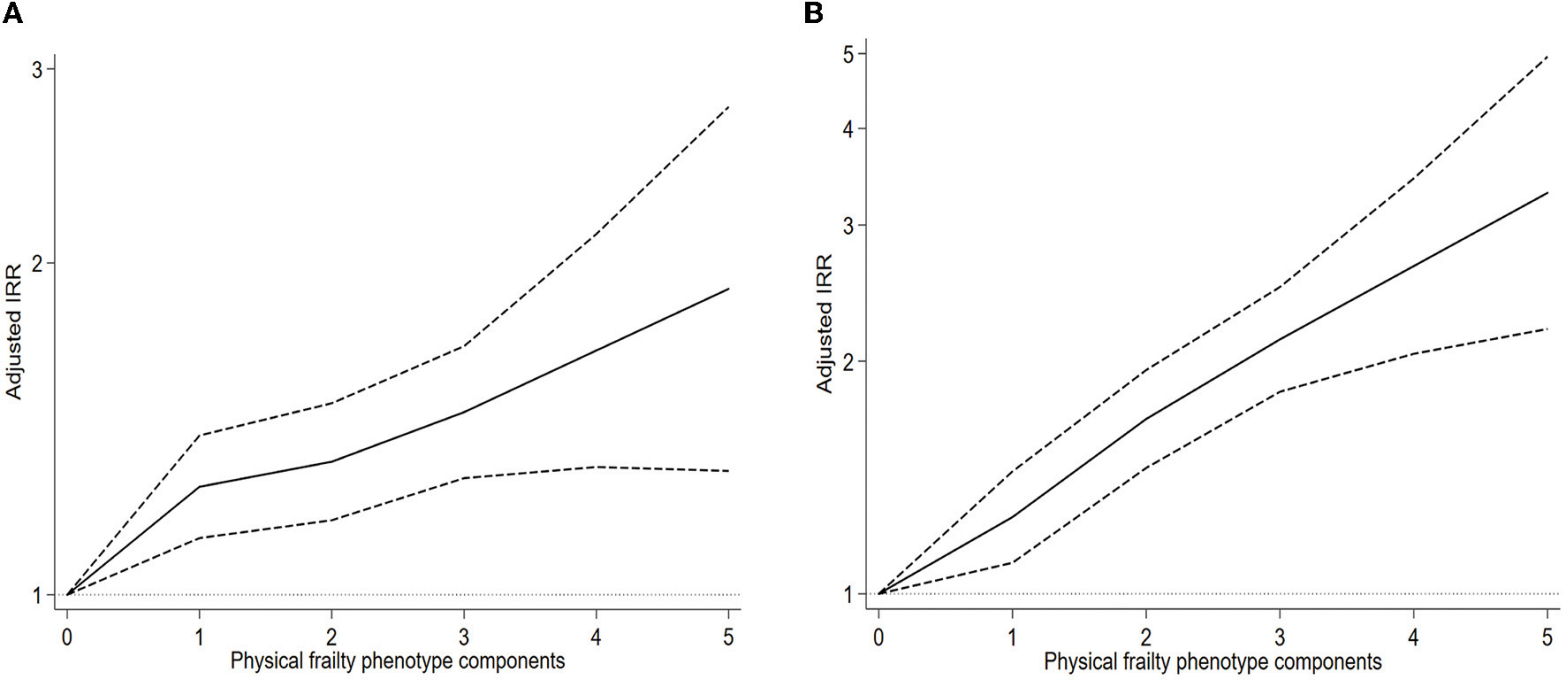
Association between frailty and health service use using restricted cubic spline regression. Graphs show the IRR for the number of outpatient visits **(A)** and inpatient hospital days **(B)** by a restricted cubic spline negative binomial model. All results were adjusted for age, gender, marital status, educational level, living residence status, socioeconomic status quartiles, health insurance type, economic development regions, smoking status, drinking frequency, and number of comorbid physical chronic non-communicable diseases. IRR, incidence rate ratio; CI, confidence interval.

A greater number of outpatient visits and inpatient hospital days were observed in the frail participants than in the robust participants (Supplementary Table 1). The results of our panel data indicated that when frailty was examined as a continuous variable, each one-component increase in frailty was associated with a higher number of outpatient visits in all models adjusting for confounders (crude model: IRR = 1.16, 95% CI = 1.11–1.22; and fully adjusted model: IRR = 1.13, 95% CI = 1.08–1.19) (Table 2). The results showed a positive association between PFP score and outpatient visits using restricted cubic spline regression (Figure 1). Compared with robust individuals, individuals with pre-frail or frail status were likely to report a higher number of outpatient visits after controlling for all covariates (pre-frail: IRR = 1.28, 95% CI = 1.16–1.41; frail: IRR = 1.45, 95% CI = 1.23–1.71). Besides, two frailty components, including weight loss and exhaustion, were significantly associated with a higher number of outpatient visits (weight loss: IRR = 1.33, 95% CI = 1.18–1.51; exhaustion: IRR = 1.44, 95% CI = 1.32–1.58) after controlling for the full list of predefined covariates (Table 2).

**Table 2:** Association between frailty and number of outpatient visits in China, 2011–2015.

**Variables**	**Number of outpatient visits, IRR (95%CI)**				
	**Model 1** ^a^	**Model 2** ^b^	**Model 3** ^c^	**Model 4** ^d^	**Model 5** ^e^
Every one-component increase in frailty	1.16 (1.11,1.22)^***^	1.17 (1.12,1.23)^***^	1.18 (1.13,1.24)^***^	1.17 (1.12,1.22)^***^	1.13 (1.08,1.19)^***^
**Frailty phenotype**					
Robust	ref	ref	ref	ref	ref
Pre-frail	1.34 (1.21,1.48)^***^	1.34 (1.21,1.48)^***^	1.35 (1.22,1.50)^***^	1.34 (1.21,1.48)^***^	1.28 (1.16,1.41)^***^
Frail	1.58 (1.35,1.86)^***^	1.61 (1.37,1.90)^***^	1.67 (1.41,1.96)^***^	1.61 (1.37,1.90)^***^	1.45 (1.23,1.71)^***^
**Frailty phenotype components**					
Weight loss (ref: no weight loss)	1.43 (1.26,1.62)^***^	1.43 (1.26,1.62)^***^	1.42 (1.25,1.61)^***^	1.41 (1.24,1.59)^***^	1.33 (1.18,1.51)^***^
Exhaustion (ref: no exhaustion)	1.57 (1.44,1.72)^***^	1.56 (1.42,1.71)^***^	1.56 (1.42,1.71)^***^	1.54 (1.41,1.69)^***^	1.44 (1.32,1.58)^***^
Low physical activity (ref: no inactivity)	0.90 (0.81,1.01)	0.91 (0.80,1.01)	0.92 (0.82,1.03)	0.92 (0.82,1.03)	0.92 (0.82,1.03)
Slowness (ref: no slowness)	1.08 (0.97,1.2)	1.08 (0.97,1.21)	1.08 (0.97,1.21)	1.06 (0.95,1.19)	1.05 (0.94,1.17)
Weakness (ref: no weakness)	1.07 (0.95,1.19)	1.09 (0.97,1.22)	1.11 (0.99,1.24)	1.08 (0.96,1.21)	1.03 (0.92,1.16)

a Model 1 was a crude model without adjustment for any covariates.

b Model 2 was adjusted for age and gender.

c Model 3 was adjusted for age, gender, marital status, educational level, living residence status, socioeconomic status quartiles, health insurance type, and economic development regions.

d Model 4 was adjusted for age, gender, marital status, educational level, living residence status, socioeconomic status quartiles, health insurance type, economic development regions, smoking status, and drinking frequency.

e Model 5 was adjusted for age, gender, marital status, educational level, living residence status, socioeconomic status quartiles, health insurance type, economic development regions, smoking status, drinking frequency, and the number of chronic non-communicable diseases.

*** p < 0.001. IRR, incidence rate ratio; CI, confidence interval.

Our results also indicated that each one-component increase in frailty was associated with a higher number of days spent in the hospital as an inpatient in all models adjusting for confounders (crude model: IRR = 1.33, 95% CI = 1.27–1.40; and fully adjusted model: IRR = 1.29, 95% CI = 1.22–1.36) (Table 3). The results showed a positive association between the PFP score and days spent in the hospital as an inpatient, using restricted cubic spline regression (Figure 1). Individuals with pre-frail or frail status were likely to report a higher number of inpatient hospital days than robust individuals after controlling for all covariates (pre-frail: IRR = 1.40, 95% CI = 1.24–1.58; frail: IRR = 2.17, 95% CI = 1.81–2.60). All the five frailty components were found to be significantly associated with a higher number of inpatient hospital days (weight loss: IRR = 1.64, 95% CI = 1.42–1.88; exhaustion: IRR = 1.43, 95% CI = 1.28–1.59; low physical activity: IRR = 1.20, 95% CI = 1.05–1.36; slowness: IRR = 1.19, 95% CI = 1.05–1.35; and weakness: IRR = 1.35, 95% CI = 1.19–1.53) after controlling for the full list of predefined covariates (Table 3).

**Table 3:** Association between frailty and number of inpatient hospital days in China, 2011–2015.

**Variables**	**Number of inpatient hospital days, IRR (95%CI)**				
	**Model 1** ^a^	**Model 2** ^b^	**Model 3** ^c^	**Model 4** ^d^	**Model 5** ^e^
Every one-component increase in frailty	1.33 (1.27,1.40)^***^	1.32 (1.25,1.38)^***^	1.37 (1.3,1.44)^***^	1.35 (1.28,1.42)^***^	1.29 (1.22,1.36)^***^
**Frailty phenotype**					
Robust	ref	ref	ref	ref	ref
Pre-frail	1.46 (1.29,1.65)^***^	1.44 (1.27,1.62)^***^	1.52 (1.35,1.73)^***^	1.50 (1.32,1.70)^***^	1.40 (1.24,1.58)^***^
Frail	2.44 (2.06,2.90)^***^	2.31 (1.94,2.76)^***^	2.61 (2.18,3.13)^***^	2.47 (2.06,2.96)^***^	2.17 (1.81,2.60)^***^
**Frailty phenotype components**					
Weight loss (ref: no weight loss)	1.87 (1.63,2.15)^***^	1.84 (1.6,2.12)^***^	1.78 (1.55,2.05)^***^	1.77 (1.53,2.03)^***^	1.64 (1.42,1.88)^***^
Exhaustion (ref: no exhaustion)	1.49 (1.34,1.66)^***^	1.51 (1.36,1.68)^***^	1.60 (1.44,1.79)^***^	1.57 (1.41,1.75)^***^	1.43 (1.28,1.59)^***^
Low physical activity (ref: no inactivity)	1.12 (0.98,1.27)	1.10 (0.97,1.25)	1.17 (1.03,1.34)^*^	1.18 (1.03,1.34)^**^	1.20 (1.05,1.36)^**^
Slowness (ref: no slowness)	1.29 (1.14,1.45)^***^	1.22 (1.07,1.38)^**^	1.27 (1.12,1.44)^***^	1.22 (1.08,1.39)^**^	1.19 (1.05,1.35)^**^
Weakness (ref: no weakness)	1.53 (1.36,1.72)^***^	1.44 (1.27,1.63)^***^	1.51 (1.34,1.71)^***^	1.44 (1.27,1.63)^***^	1.35 (1.19,1.53)^***^

a Model 1 was a crude model without adjustment for any covariates.

b Model 2 was adjusted for age and gender.

c Model 3 was adjusted for age, gender, marital status, educational level, living residence status, socioeconomic status quartiles, health insurance type, and economic development regions.

d Model 4 was adjusted for age, gender, marital status, educational level, living residence status, socioeconomic status quartiles, health insurance type, economic development regions, smoking status, and drinking frequency.

e Model 5 was adjusted for age, gender, marital status, educational level, living residence status, socioeconomic status quartiles, health insurance type, economic development regions, smoking status, drinking frequency, and the number of chronic non-communicable diseases.

* p < 0.05,

** p < 0.01,

*** p < 0.001. IRR, incidence rate ratio; CI, confidence interval.

### 3.3. Stratification analyses

**Figure 2:**
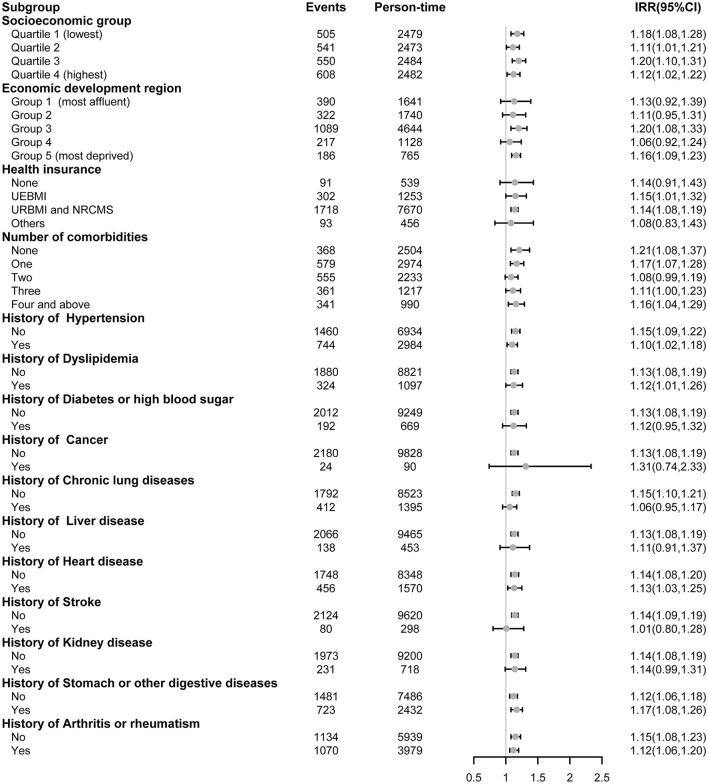
Association between every one-component increase in frailty and the number of outpatient visits stratified by socioeconomic group, health insurance, and physical comorbidities. The panel-data random-effects negative binomial regression models were used for analyses and adjusted for other covariates with the stratification variable removed. IRR refers to the incidence rate ratio. UEBMI refers to urban employee basic medical insurance. URBMI refers to urban resident basic medical insurance. NRCMS refers to the new rural cooperative medical scheme. Others include government medical insurance, private medical insurance, and other medical insurances.

**Figure 3:**
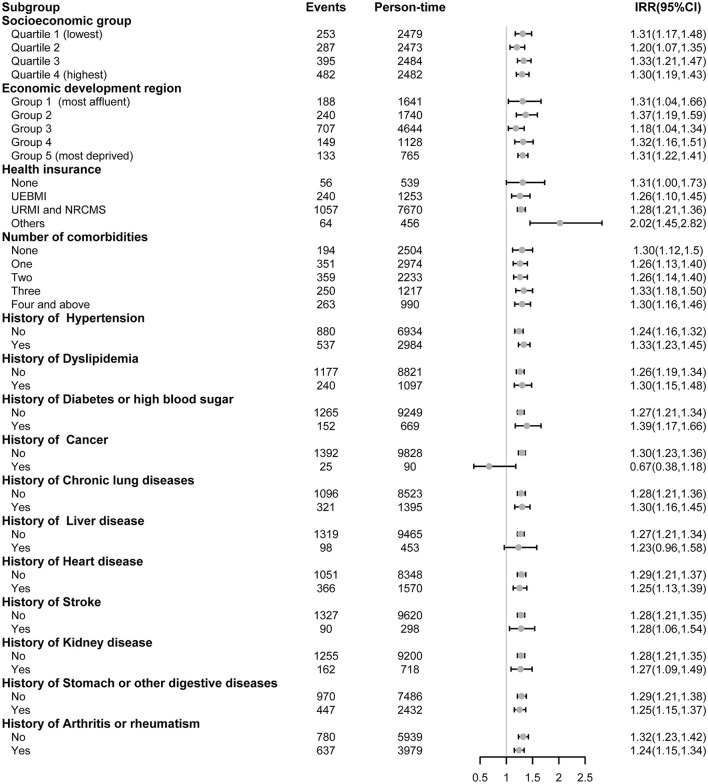
Association between every one-component increase in frailty and inpatient hospital days stratified by socioeconomic group, health insurance, and physical comorbidities. The panel-data random-effects negative binomial regression models were used for analyses and adjusted for other covariates with the stratification variable removed. IRR refers to the incidence rate ratio. UEBMI refers to urban employee basic medical insurance. URBMI refers to urban resident basic medical insurance. NRCMS refers to the new rural cooperative medical scheme. Others include government medical insurance, private medical insurance, and other medical insurances.

The results of the stratification analyses that examined whether the effect of each elevated component of frailty on the number of outpatient visits and inpatient hospital days varied by socioeconomic status, health insurance type, number of comorbidities, and history of each chronic non-communicable disease are shown in Figures 2, 3, respectively. The results showed that each component increase in frailty was associated with a greater number of outpatient visits across all socioeconomic status groups in the most deprived economic development regions and the total number of physical comorbidity groups (except for three types of physical comorbidities) (Figure 2). The associations between each component increase in frailty and the number of outpatient visits were similar across all socioeconomic statuses, economic development regions, health insurance types, and the total number of physical comorbidities. For each chronic non-communicable disease, each component increase in frailty was associated with a greater number of inpatient hospital days (except for a history of cancer and liver diseases) (Figure 3).

### 3.4. Sensitivity analysis

To test the robustness of our results, we repeated our analysis using a Poisson regression model with a robust standard error to evaluate the association among frailty status, outpatient visits, and inpatient hospital days. The results of the Poisson regression with a robust standard error were consistent with our findings of the negative binomial regression, showing that frailty status was associated with an increased number of outpatient visits and inpatient hospital days (Supplementary Table 2).

## 4. Discussion

This panel data study analyzed the effect of frailty on health service use in Chinese individuals aged 60 years and older. Our findings indicate that frailty status is associated with increased outpatient visits and inpatient hospital days. The presence of two frailty components (weight loss and exhaustion) was independently associated with increased outpatient visits after controlling for several confounders, and all five frailty components (weight loss, exhaustion, low physical activity, slowness, and weakness) were independently associated with increased inpatient hospital days.

A previous longitudinal analysis of 95,863 patients in England indicated that, compared to robust individuals, severely frail individuals had a higher rate of annual general practitioner consultation, increased emergency hospital admissions, and longer inpatient hospital days (26). Another study of 701 older adults in Singapore found that, compared with robust individuals, frail individuals reported more specialized outpatient clinic visits, emergency department visits, day surgery attendances, and hospitalisations (27). A sample of 1,060 participants from the Helsinki Birth Cohort Study suggested that frailty was associated with the number of inpatient hospital days, emergency visits, and hospital admissions (28). As expected, after controlling for several confounding factors, frailty status was associated with increased health service use in our study, and this association was significant regardless of whether every one-component increase in frailty or robust, pre-frail, and frail definitions were used to assess frailty status. The link between frailty and increased health service use may be related to a weakened immune system, endocrine system, brain, and skeletal muscles in frail individuals (4, 29). Frailty is an age-related consequence of the decline in multiple physiological systems, which is associated with atypical and complex illness conditions and leads to sharp changes in health conditions triggered by relatively stressful events (4). Among frail older patients, the increase in healthcare use, particularly inpatient care, could be attributed to the co-occurrence of other geriatric syndromes associated with frailty, such as impaired quality of life, loneliness (30), delirium (31, 32), recurrent falls (33), and reduced mobility (34). In addition, comprehensive multidisciplinary management of frailty is important in clinical practice. However, the hidden incidence and low awareness of frailty may result in a disease-specific care model rather than a multidisciplinary comprehensive care model, and unfavorable treatment and an undesirable prognosis may make frail individuals repeatedly seek healthcare (35).

Although the association between frailty and health service use has been widely examined, the contribution of specific components of frailty to health service use remains unclear. Our study found that weight loss and exhaustion were independently associated with increased outpatient visits, and all five frailty components (weight loss, exhaustion, low physical activity, slowness, and weakness) were independently associated with longer inpatient hospital days. Unintentional weight loss is a common problem in older adults and may be a sign of a medical condition, such as oral disorders, non-malignant gastrointestinal diseases, psychiatric disorders, or cancer (36, 37). Several social factors, such as financial constraints and isolation, may also contribute to unintentional weight loss (38). Unintentional weight loss may also complicate the treatment and recovery from medical conditions (38), resulting in increased health service use. Exhaustion is a state of emotional, mental, and physical health caused by prolonged stress (39, 40). It is considered the core dimension of burnout (41). It can hinder an individual's ability to recover and is associated with severe health consequences. Compared to those without exhaustion, individuals who reported experiencing mild and severe exhaustion might have greater impairments in emotional, physical, and social functioning and increased resource utilization (41).

In our study, an increased number of outpatient visits was associated with each component increase in frailty among individuals from relatively deprived economic development regions, which indicates that targeted prevention and intervention of frailty are important to reduce the health service burden, particularly for individuals from low socioeconomic groups or those living in deprived economic development regions. Our study also indicated that each component increase in frailty was associated with a greater number of outpatient visits and inpatient hospital days across all physical comorbidity groups. However, the association between each component's increase in frailty and outpatient visits was not significant among individuals with diabetes or high blood sugar, cancer or malignant tumors, chronic lung diseases, liver disease (except fatty liver, tumors, and cancer), stroke, and kidney disease. The underlying reason might be that individuals who experienced the aforementioned comorbid chronic diseases required periodic physical examinations in outpatient care (42), regardless of whether they were frail or not. Periodic outpatient visits due to chronic diseases may conceal or attenuate frailty. Our study also found that each component increase in frailty was associated with inpatient hospital days, which revealed the adverse effects of frailty on health service use.

This study has several implications for public health. Frailty is a common phenomenon among older adults and is associated with a progressively greater probability of higher health-service use. Screening for frailty status should be included in clinical practice to identify vulnerable individuals and provide proper action to counter frailty and its adverse effects, specifically for individuals with physical comorbidities, from low socioeconomic groups, or living in deprived economic development regions. The study findings also indicate that there is a need for comprehensive interventions, such as exercise interventions, nutritional interventions, and pharmacological agents, to reduce the development and progression of frailty and alleviate the economic impacts of frailty on individuals and health systems in China.

The strengths of this study include the large sample size, nationally representative longitudinal survey, the inclusion of specific frail components, focus on discrete health service areas, such as outpatient visits and days of hospitalization, and subgroup analyses. However, this study had some limitations that must be noted. First, the numbers of outpatient visits and inpatient hospital days were collected using a self-reported questionnaire instead of electronic medical records. This approach was not precise, and there was recall bias in our research. Second, each chronic disease group included a range of diseases, and individuals were asked to indicate whether they had at least one disease in each chronic disease group based on their clinical diagnosis. The specific types of disease in each chronic disease group were not collected. Third, information on healthcare costs for outpatient visits or hospitalisations and other health service utilization indicators such as accidents and emergency department visits, prescriptions, referrals, immunisations, and screening were not collected in the current study. Future studies should evaluate additional health service utilization indicators to verify the effects of frailty.

## 5. Conclusion

In conclusion, the results of this study suggest that frailty is associated with a significant increase in the use of health services. The effect of frailty on inpatient hospital days persisted in different socioeconomic groups across all health insurance programmes and physical commonalities. Frailty is a risk indicator with the potential to drive health service use. Improved recognition and intervention for frailty in older individuals might have an important impact on reducing overall health service use, given the rapidly aging population in China.

## Data availability statement

The original contributions presented in the study are included in the article/Supplementary material, further inquiries can be directed to the corresponding author.

## Ethics statement

The studies involving human participants were reviewed and approved by the Biomedical Ethics Review Committee of Peking University (No: IRB00001052-11015). The patients/participants provided their written informed consent to participate in this study.

## Author contributions

RY and JZ conceived and designed the study. RY and XD conducted the initial analysis and analyzed the literature. RY, LL, and XD interpreted the data. RY wrote the first draft of the report. RY, LL, XD, and JZ provided advice on the first draft and revised the report critically for important intellectual content. All authors reviewed and provided final approval of the submitted and published versions.
