# Supplementary material for: Association of frailty with health service use among older Chinese adults: analysis of population-based panel data
Source: Front Public Health. 2023 Jul 28;11:1011588. doi: 10.3389/fpubh.2023.1011588 (PMC10420091; doi:10.3389/fpubh.2023.1011588)
Supplement: Supplementary file 1 [file Table_1.docx]

Supplementary Material

# Supplementary Data

**Table S1: Distribution of number of outpatient visits and inpatient hospital days by socio-demographic characteristics and frailty phenotype (pooled panel survey data for 2011-15).**

| **Characteristics** | **N** |  | **Number of outpatient visits** | | |  | **Inpatient hospital days** | | |
| --- | --- | --- | --- | --- | --- | --- | --- | --- | --- |
|  |  |  | Mean | SD | P |  | Mean | SD | P |
| All participants | 9918 |  | 0.50 | 1.46 |  |  | 2.46 | 10.01 |  |
| Age (years) |  |  |  |  |  |  |  |  |  |
| 60-70 | 6348 |  | 0.53 | 1.55 |  |  | 2.17 | 9.24 |  |
| 70-80 | 3112 |  | 0.44 | 1.18 |  |  | 2.90 | 11.09 |  |
| 80 and above | 458 |  | 0.54 | 1.74 | 0.015 |  | 3.41 | 12.20 | <0.001 |
| Gender |  |  |  |  |  |  |  |  |  |
| Male | 4581 |  | 0.57 | 1.63 |  |  | 2.19 | 9.14 |  |
| Female | 5337 |  | 0.44 | 1.28 | <0.001 |  | 2.69 | 10.70 | 0.013 |
| Education |  |  |  |  |  |  |  |  |  |
| No formal education/illiterate | 2958 |  | 0.49 | 1.44 |  |  | 2.12 | 9.67 |  |
| Primary school | 5019 |  | 0.50 | 1.43 |  |  | 2.54 | 10.64 |  |
| Middle school and above | 1941 |  | 0.50 | 1.54 | 0.913 |  | 2.76 | 8.76 | 0.063 |
| Marital status |  |  |  |  |  |  |  |  |  |
| Married and partnered | 7974 |  | 0.49 | 1.43 |  |  | 2.45 | 9.75 |  |
| Unmarried and others | 1944 |  | 0.54 | 1.57 | 0.124 |  | 2.49 | 11.04 | 0.859 |
| Socioeconomic group |  |  |  |  |  |  |  |  |  |
| Quartile 1 (lowest) | 2479 |  | 0.43 | 1.24 |  |  | 1.58 | 8.86 |  |
| Quartile 2 | 2473 |  | 0.47 | 1.29 |  |  | 1.59 | 6.27 |  |
| Quartile 3 | 2484 |  | 0.50 | 1.46 |  |  | 2.85 | 11.46 |  |
| Quartile 4 (highest) | 2482 |  | 0.59 | 1.77 | 0.001 |  | 3.80 | 12.18 | <0.001 |
| Residence status |  |  |  |  |  |  |  |  |  |
| Urban | 3065 |  | 0.49 | 1.51 |  |  | 2.97 | 9.97 |  |
| Rural | 6853 |  | 0.50 | 1.43 | 0.577 |  | 2.23 | 10.02 | <0.001 |
| Economic development region |  |  |  |  |  |  |  |  |  |
| Group 1 (most affluent) | 542 |  | 0.43 | 1.16 |  |  | 2.63 | 9.59 |  |
| Group 2 | 1041 |  | 0.37 | 1.00 |  |  | 2.53 | 8.79 |  |
| Group 3 | 2044 |  | 0.45 | 1.52 |  |  | 2.13 | 10.10 |  |
| Group 4 | 1068 |  | 0.53 | 1.88 |  |  | 3.37 | 10.52 |  |
| Group 5 (most deprived) | 5223 |  | 0.54 | 1.43 | 0.002 |  | 2.37 | 10.14 | 0.020 |
| Health insurance |  |  |  |  |  |  |  |  |  |
| None | 539 |  | 0.37 | 1.32 |  |  | 1.84 | 7.27 |  |
| Urban Employee Basic Medical Insurance | 1253 |  | 0.51 | 1.73 |  |  | 3.94 | 11.75 |  |
| Urban Resident Basic Medical Insurance and New Rural Cooperative Medical Scheme | 7670 |  | 0.51 | 1.43 |  |  | 2.23 | 9.67 |  |
| Others | 456 |  | 0.41 | 1.14 | 0.073 |  | 2.98 | 12.55 | <0.001 |
| Smoking status |  |  |  |  |  |  |  |  |  |
| Never | 5312 |  | 0.54 | 1.54 |  |  | 2.42 | 9.37 |  |
| Former | 1211 |  | 0.56 | 1.37 |  |  | 4.77 | 16.64 |  |
| Current | 3395 |  | 0.41 | 1.34 | <0.001 |  | 1.69 | 7.36 | <0.001 |
| Drinking frequency |  |  |  |  |  |  |  |  |  |
| Never | 6670 |  | 0.56 | 1.57 |  |  | 2.86 | 11.17 |  |
| <1/month | 646 |  | 0.46 | 1.27 |  |  | 2.13 | 8.41 |  |
| >=once/month | 2602 |  | 0.35 | 1.15 | <0.001 |  | 1.51 | 6.60 | <0.001 |
| Number of comorbidities |  |  |  |  |  |  |  |  |  |
| None | 2504 |  | 0.32 | 1.32 |  |  | 1.30 | 6.51 |  |
| One | 2974 |  | 0.40 | 1.21 |  |  | 2.11 | 11.01 |  |
| Two | 2233 |  | 0.55 | 1.44 |  |  | 2.64 | 10.73 |  |
| Three | 1217 |  | 0.73 | 1.80 |  |  | 3.27 | 9.59 |  |
| Four and above | 990 |  | 0.86 | 1.84 | <0.001 |  | 5.02 | 12.22 | <0.001 |
| Frailty phenotype |  |  |  |  |  |  |  |  |  |
| Robust | 3455 |  | 0.38 | 1.20 |  |  | 1.63 | 6.75 |  |
| Pre-frail | 5625 |  | 0.54 | 1.52 |  |  | 2.58 | 10.07 |  |
| Frail | 838 |  | 0.70 | 1.90 | <0.001 |  | 5.08 | 17.58 | <0.001 |

SD, Standard deviations. UEBMI, Urban Employee Basic Medical Insurance; URBMI, Urban Resident Basic Medical Insurance; NRCMS, New Rural Cooperative Medical Scheme; Others, government, private, and other medical insurances.

**Table S2: Association between frailty status, outpatient visits and inpatient hospital days analyzed by Poisson regression models** **with robust standard error.**

| **Variables** | **Number of outpatient visits** | |  | **Number of inpatient hospital days** | |
| --- | --- | --- | --- | --- | --- |
|  | IRR (95%CI) | P |  | IRR (95%CI) | P |
| Every one-component increase in frailty | 1.12(1.08,1.16) | <0.001 |  | 1.20(1.17,1.22) | <0.001 |
| Frailty phenotype |  |  |  |  |  |
| Robust | ref |  |  | ref |  |
| Pre-frail | 1.24(1.15,1.35) | <0.001 |  | 1.30(1.24,1.35) | <0.001 |
| Frail | 1.51(1.33,1.72) | <0.001 |  | 1.86(1.74,1.98) | <0.001 |
| Frailty phenotype components |  |  |  |  |  |
| Weight loss(ref: no weight loss) | 1.26(1.15,1.39) | <0.001 |  | 1.42(1.35,1.48) | <0.001 |
| Exhaustion (ref: no exhaustion) | 1.27(1.17,1.36) | <0.001 |  | 1.21(1.16,1.26) | <0.001 |
| Inactivity (ref: no inactivity) | 0.93(0.86,1.01) | 0.093 |  | 1.05(1.01,1.09) | 0.025 |
| Slowness (ref: no slowness) | 1.06(0.97,1.15) | 0.196 |  | 1.03(0.99,1.07) | 0.193 |
| Weakness(ref: no weakness) | 1.15(1.05,1.26) | 0.002 |  | 1.45(1.39,1.52) | <0.001 |

All results were adjusted for age, gender, marital status, educational level, living residence status, socioeconomic status quartiles, health insurance type, economic development regions, smoking status, drinking frequency, and the number of chronic non-communicable diseases.IRR, incidence

rate ratio; CI, confidence interval.
